# White matter abnormalities in paediatric obsessive–compulsive disorder: a systematic review of diffusion tensor imaging studies

**DOI:** 10.1007/s11682-023-00761-x

**Published:** 2023-03-20

**Authors:** Maryam Haghshomar, Seyed Peyman Mirghaderi, Parnian Shobeiri, Anthony James, Mojtaba Zarei

**Affiliations:** 1 The Medical School, Tehran University of Medical Sciences, Tehran, Iran; 2 Highfield Family and Adolescent Unit, Warneford Hospital, Department of Psychiatry, University of Oxford, Oxford, UK; 3 Institute of Medical Science and Technology, Shahid Beheshti University, Tehran, Iran; 4 Departments of Neurology, Odense University Hospital, Odense, Denmark; 5 Department of Clinical Research, University of Southern Denmark, Odense, Denmark

**Keywords:** Diffusion tensor imaging, Obsessive–compulsive disorder, White matter, Paediatric

## Abstract

Microstructural alterations in white matter are evident in obsessive–compulsive disorder (OCD) both in adult and paediatric populations. Paediatric patients go through the process of maturation and thus may undergo different pathophysiology than adult OCD. Findings from studies in paediatric obsessive–compulsive disorder have been inconsistent, possibly due to their small sample size or heterogeneous populations. The aim of this review is to provide a comprehensive overview of white matter structures in paediatric obsessive–compulsive disorder and their correlation with clinical features. Based on PRISMA guidelines, we performed a systematic search on diffusion tensor imaging studies that reported fractional anisotropy, mean diffusivity, radial diffusivity, or axial diffusivity alterations between paediatric patients with obsessive–compulsive disorder and healthy controls using voxel-based analysis, or tract‐based spatial statistics. We identified fifteen relevant studies. Most studies reported changes predominantly in the corpus callosum, cingulum, arcuate fasciculus, uncinate fasciculus, inferior longitudinal fasciculus, superior longitudinal fasciculus, inferior fronto-occipital fasciculus, corticospinal tract, forceps minor and major, and the cerebellum in paediatric obsessive–compulsive disorder. These alterations included increased and decreased fractional anisotropy and radial diffusivity, and increased mean and axial diffusivity in different white matter tracts. These changes were associated with obsessive–compulsive disorder symptoms. Moreover, specific genetic polymorphisms were linked with cerebellar white matter changes in paediatric obsessive–compulsive disorder. White matter changes are widespread in paediatric OCD patients. These changes are often associated with symptoms however there are controversies in the direction of changes in some tracts.

**Supplementary Information:**

The online version contains supplementary material available at 10.1007/s11682-023-00761-x.

## Introduction

Obsessive–compulsive disorder (OCD) is the fourth most common mental disorder with a lifetime prevalence of approximately 2–3%. OCD is characterized by recurrent obsessive thoughts and intrusions (obsessions) and habitual behaviours (compulsions). These symptoms disturb patients’ daily activity and affect their quality of life (Drubach, 2015; Ferreira et al., 2020). Around 1–3% of children experience OCD symptoms and unlike adult OCD, boys are more commonly affected by the symptoms than girls. The mean age of paediatric OCD is ten years but symptoms may appear in children as young as five years old (Mataix-Cols et al., 2008).

**Fig. 1:**
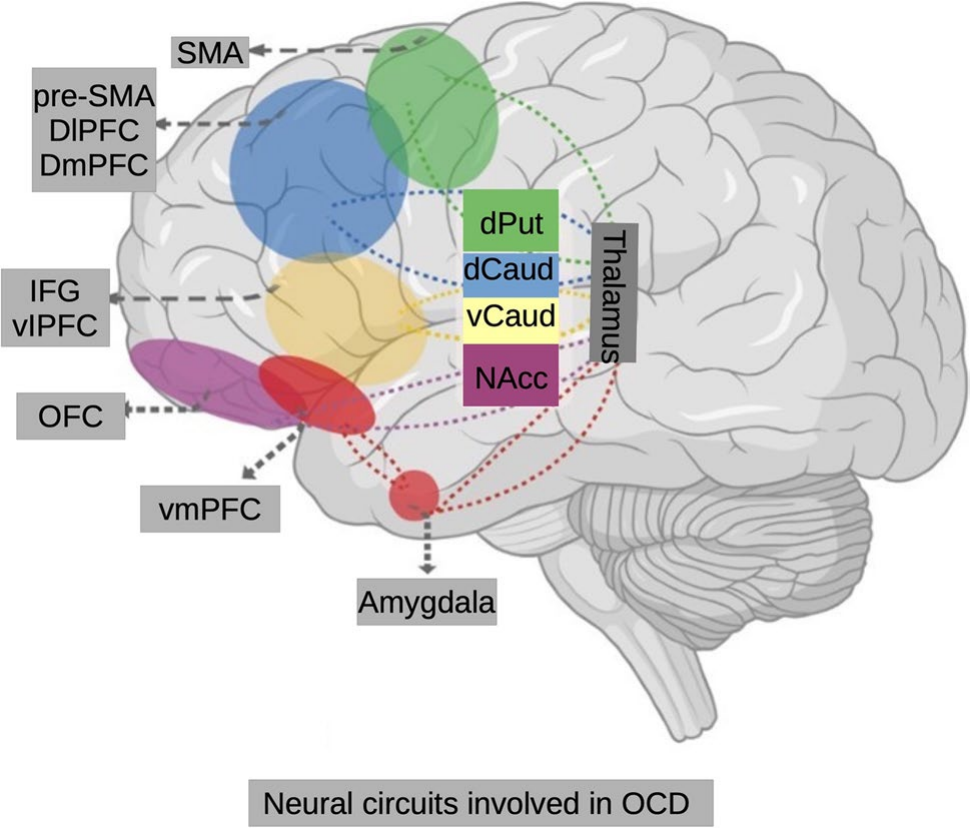
Neural circuits involved in OCD. Abbreviations: SMA=Supplementary motor area, DlPFC=dorsolateral prefrontal cortex, DmPFC=Dorsomedial prefrontal cortex, IFG=Inferior frontal gyrus, vlPFC=Ventromedial prefrontal cortex, PFC=Orbitofrontal cortex, vmPFC=Ventromedial prefrontal cortex, dPut=dorsal putamen, dCaud=Dorsal caudate, vCaud=Ventral caudate, NAcc=Nucleus accumbens

The aetiology of OCD has been linked to various brain systems. Earlier hypotheses were based on clinical observations from pallidal and frontal lobe lesions (Eslinger & Damasio, 1985) but data obtained from brain imaging in the last three decades revolutionized our knowledge of underlying neurobiology. The current models are focused on the orbitofronto-striatal circuit (Stein, 2002). This circuit includes medial orbitofrontal, anterior cingulate and temporolimbic cortices, striatum, and thalamus. The anterior cingulate cortex, the dorsolateral prefrontal cortex, and the orbitofrontal cortex seem to be more relevant to the psychopathology of OCD (Chamberlain et al., 2008) (Fig. 1). However, recent neuroimaging studies in OCD suggest that this model may not be sufficient to explain the diverse clinical manifestations of OCD. A broader model highlights involvements of other structures such as dorsolateral prefronto-striatal circuit (dorsomedial, dorsolateral, ventrolateral, and frontopolar prefrontal cortices), and reciprocally connected temporo-parieto-occipital associative areas. The notion indicates network pathology rather than a specific anatomical one. Alternatively, OCD may be a heterogenous condition with various neural pathology but common psychiatric symptoms (Menzies et al., 2008a, b; Piras et al., 2015).

## Diffusion tensor imaging and white matter alterations

Diffusion tensor imaging (DTI) allows the assessment of WM integrity within the brain major tracts. The method has been applied to study white matter changes in the OCD (Correia et al., 2008; Piras et al., 2021a, b, c). DTI has been widely used in neurodevelopmental (Abdolalizadeh et al., 2021; Ghazi Sherbaf et al., 2019; Piras et al., 2021a, b, c; Piras et al., 2013) and neurocognitive studies too (Seyedmirzaei et al., 2022).

The principle of DTI is the measurement of diffusion of water in the brain tissue using changes in the radiofrequency signal that occurs as water molecules move towards or away from the source of radiofrequency. Water randomly diffuses in all directions in an unbounded environment. However, in the WM the direction of water diffusivity is mainly aligned with neural pathways as axonal membranes and myelin limit its radial diffusivity.

There are four main parameters often used as surrogate markers of diffusion in DTI studies. These markers indirectly represent microstructure of neural fibers in the brain. Fractional anisotropy (FA) is the most common indicator among these and represents the degree of anisotropy, which in turn is computed from the eigenvalues of the diffusion tensors in each of the axes. FA is particularly affected by density, orientation, WM integrity and myelination in each voxel. It decreases in neural pathology that affects myelination and orientation of the fibres. The highest FA values are found in corpus callosum and internal capsule, and the lowest values are in the grey matter and in voxels containing crossing fibers (DeBoy et al., 2007). The FA values in dense and well-aligned WM tracts are higher, whereas the FA values in CSF and damaged fibers are lower (Smith et al., 2006). Determination of FA and calculation of eigenvectors are the principle upon which DT tractography is modelled. Axial diffusivity (AD) refers to the diffusion along the main axis (principal eigenvector) of the diffusion model, and radial diffusivity (RD) refers to the diffusion vector perpendicular to the main eigenvector, which is calculated as the mean of secondary and tertiary eigenvectors (Song et al., 2003). Studies in mice showed that AD reflects axonal damage, whereas RD is affected by myelination (Frydman et al., 2016; Song et al., 2003). These two parameters particularly change with aging (Bennett et al., 2010). Mean diffusivity (MD) is the average magnitude of diffusivity along the xyz directions. MD is a non-specific, but sensitive, metric and is influenced by any condition which restricts diffusion of water freely (Bosch et al., 2012). DTI resolution does not allow imaging at the axonal level but provides information that is relevant to neural tract anatomy usually at a 2–3 mm^3^ scale.

There are different approaches to the analysis of DTI data in order to assess WM microstructure. Voxel-based morphometry (VBM) is probably the most common approach. In this method, diffusion data is aligned with a high-resolution template, which is spatially normalized and smoothened prior to running statistical tests. As a result, the outcomes are highly reliant on a variety of parameters, such as the accuracy of the registration, smoothing filter size, etc. (Jones et al., 2005). Tract-based spatial statistics (TBSS) is another approach (Smith et al., 2006) that is more robust than VBM. In this method, each individual's FA data is projected onto a skeleton, which is constructed from the center of major white matter tracts. This will substantially remove the potential errors, which may arise from registration and partial volume effect. The analysis of DTI data by region of interest (ROI) is another frequently utilized approach. In this approach, regions are defined using an atlas, or by automatic or manual segmentation of the ROI. Various DTI measures can then be calculated. Structural connectivity between different brain regions can be characterized non-invasively and in vivo, by using a fibre-tracking algorithm. The stepwise production of streamlines is the foundation of the most used fibre-tracking technique. Using this technique, it is not possible to differentiate between some scenarios solely at the voxel level since varied local fibre geometries, such as crossing, kissing, bending, and fanning, might result in the same MRI data (Jeurissen et al., 2019).

## Neural basis of OCD

Neuroimaging findings in OCD strongly suggest the involvement of “affective” fronto-striatal loop, which comprises of orbitofrontal cortex, anterior cingulate, striatum, thalamus, and temporolimbic regions (Menzies et al., 2008a, b). Functional studies suggest dysfunction in the network responsible for action selection based on the associated reward, which again involves orbitofrontal cortex, anterior cingulate cortex, rostral cingulate motor area, and motor cortex (Menzies et al., 2008a, b). “Executive” dorsolateral prefronto-striatal circuit, is also involved in OCD. This circuit includes frontal regions like dorsolateral, ventrolateral, and frontopolar prefrontal cortex and more posterior parts of the brain like temporal, parietal, and occipital lobes. Adjacent WM alterations were observed in these regions (Menzies et al., 2008a, b; Piras et al., 2015).

In a large study of cortical morphometry in OCD (Boedhoe et al., 2018) authors found that the surface area for the transverse temporal cortex was significantly decreased in adult OCD compared to healthy controls. They also had a significantly thinner inferior parietal cortex. Medicated adult patients also displayed thinner cortices across the brain. In comparison, paediatric OCD patients had significantly thinner superior and inferior parietal cortices, and medicated OCD patients showed the lower surface area in frontal regions (Boedhoe et al., 2018).

Other meta-analyses and mega-analyses of VBM studies of OCD, showed a smaller volume of the dorsomedial prefrontal cortex, dorsal anterior cingulate cortex, and bilateral insula-operculum and a greater volume of the thalamus, cerebellum, and ventral part of the putamen (Christian et al., 2008; Van den Heuvel et al., 2022). The striatal finding, in particular, is associated with the disease duration in OCD (Weeland et al., 2022). OCD-related thalamic volume differences are driven by both age and medication status (Boedhoe et al., 2018; Van den Heuvel et al., 2022; Zarei et al., 2011).

A study by Togao and colleagues (Togao et al., 2010) designed to assess morphometry in adult OCD compared to healthy controls demonstrated that the right premotor area, right orbitofrontal cortex, right dorsolateral prefrontal cortex, bilateral temporal and occipital areas had decreased grey matter volume. Additionally, they discovered a substantial decrease in WM volume in the left anterior cingulate gyrus and a significant increase in WM volume in the right orbitofrontal region and right anterior limb of the internal capsule (Togao et al., 2010).

DTI studies of adult OCD found reduced FA in the genu and splenium of the corpus callosum, cingulum bundle, superior longitudinal fasciculus, corona radiata, and orbitofrontal WM and increased FA in connections related to amygdala and parieto-occipital area (Hu et al., 2020; Piras et al., 2021a, b, c, 2013). Additionally, patients with adult OCD showed thinner corpus callosum compared to controls and to patients with other mental disorders (Piras et al., 2021c). Higher RD in the genu and body of the corpus callosum was reported in the adult OCD (Gan et al., 2017; Magioncalda et al., 2016; Rus et al., 2017; Zhou et al., 2018). Koch et al. reviewed DTI studies on adult and paediatric OCD patients and found that WM FA is often reduced in cingulum bundle, corpus callosum, and anterior limb of the internal capsule in adult OCD. However, FA and WM connectivity were increased in paediatric OCD (Koch et al., 2014). The ENIGMA consortium (Piras et al., 2021a, b, c), conducted the largest meta-analysis of WM in OCD. They found significant FA changes in the sagittal stratum, and posterior thalamic radiation in adult OCD compared to healthy controls. Further changes including higher MD in sagittal stratum and higher RD in posterior thalamic radiation and sagittal stratum were specific to adult OCD. They did not find any detectable WM changes in paediatric OCD.

Clinical parameters known to have a meaningful association with WM microstructure changes differ between paediatric and adult patients (such as disease duration or long-term pharmaceutical therapy) (Ashraf-Ganjouei et al., 2019; Benedetti et al., 2013). These patient groups also differ from each other depending on the stage of development of white matter or myelination. During childhood and adolescence, WM anisotropy changes in brain regions responsible for attention, cognition, and motor ability. Studies showed that with aging, FA values increased in the corpus callosum, arcuate fasciculus, prefrontal regions, basal ganglia, internal capsule, thalamic pathways, and ventral visual pathways (Barnea-Goraly et al., 2005; Giorgio et al., 2010).

Paediatric OCD can be profoundly different from adult cases due to the process of brain maturation, clinical symptomatology, disease duration and effect of medication use. In this study, we aimed to systematically review DTI studies in paediatric patients with OCD to provide a comprehensive overview of WM changes in this condition.

## Methods and materials

### Eligibility criteria

DTI studies of OCD patients under the age of 18 years were included in this study. Review articles, case reports, commentaries and letters, and animal studies were excluded.

### Literature search

**Fig. 2:**
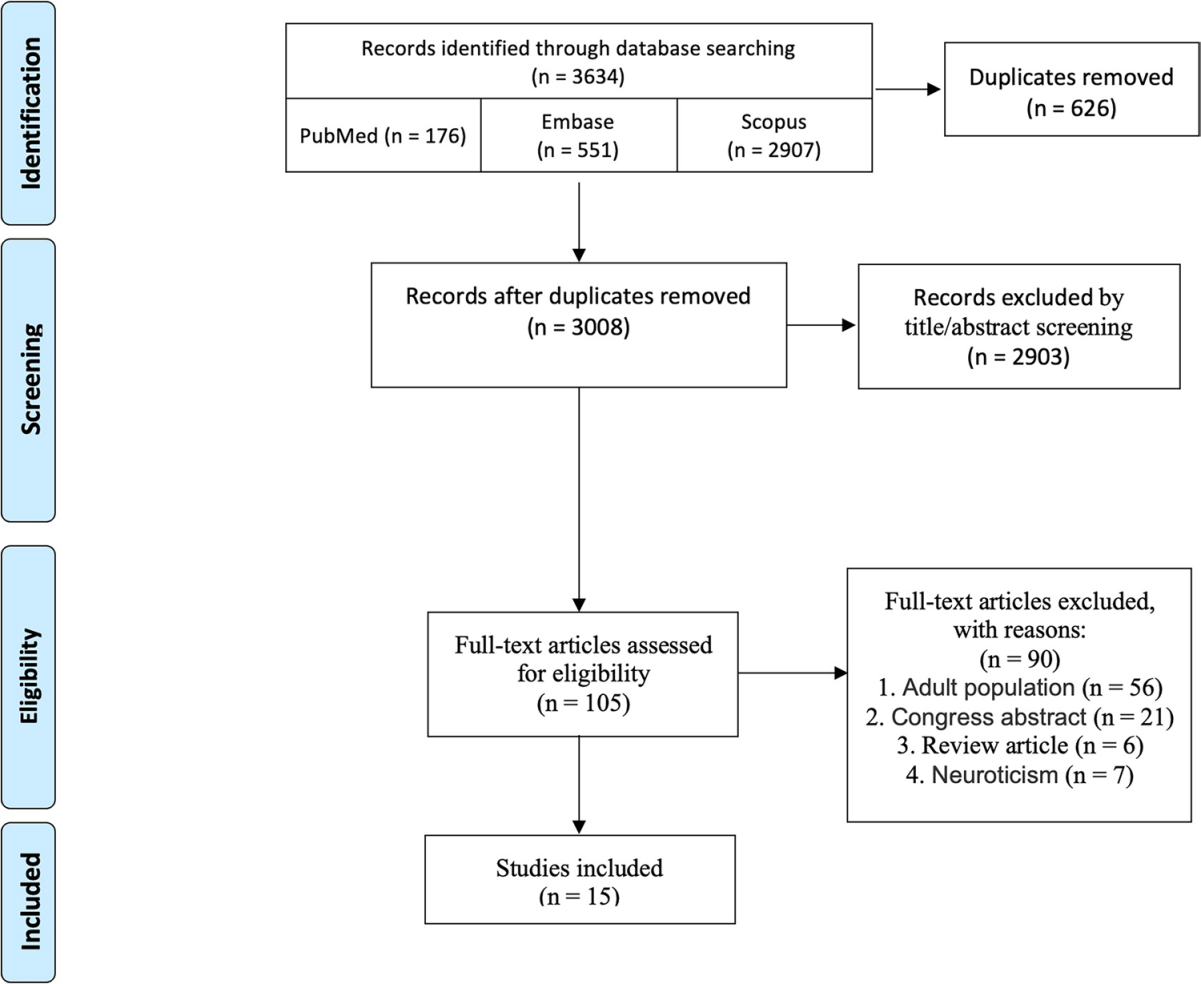
The process of screening and study selection based on the PRISMA guidelines

We performed a systematic review of the literature based on the PRISMA framework (http://www.prisma-statement.org). PubMed, EMBASE, and Scopus databases were screened till January 2022 to identify studies with the issue of DTI changes in paediatric OCD patients, applying the search term: ("Obsessive–Compulsive Disorder"[Mesh] OR "Obsessive–Compulsive Disorder" OR OCD OR "Anankastic Personality" OR "Neurosis") AND ("Diffusion Tensor Imaging"[Mesh] OR "Diffusion Tensor Imaging" OR DTI OR "diffusion MRI" OR "dMRI" OR "diffusion magnetic resonance imaging") in PubMed website. ('obsessive–compulsive disorder' OR OCD OR neurosis) AND ('Diffusion Tensor Imaging' OR DTI OR 'diffusion MRI' OR 'diffusion magnetic resonance imaging') in Embase and Scopus websites. This search was completed with no prior restrictions. Obtained results were added to the Covidence website (https://www.covidence.org). Figure 2 illustrates our process of screening and study selection based on the PRISMA guidelines.

This review was not pre-registered.

### Screening and data extraction

The screening was performed by two investigators (M.H and S.P.M). First, titles and abstracts were screened, and eligible studies were chosen for full-text screening. Then, we obtained full text of the eligible articles. Finally, paediatric OCD whole brain or ROI DTI studies with full text were included in this study. After performing data extraction, we recorded the demographic and clinical profile of participants in these studies (Table 1) and used a separate table to record the imaging modalities and findings (Table 2). Table 2 demonstrates between-group discrepancy of participants with OCD compared with healthy control participants and significant correlations between diffusivity values and symptom severity in OCD patients.

**Table 1:** Overview of reviewed articles; demographic and clinical profile of participants in DTI studies

Study	Demographic and Clinical Data															
	Study groups	N (with DTI)/males	Mean Age ± SD	Disease Duration (months)	Age of onset	IQ	Handedness R/L/both	CDI (total)	BDI Score	CY-BOCS (total)	OCD diagnosis tests	Medication (type, number and dose)	Matched in:	Differed in: *	Neuropsychological Assessments	Comorbidity
Zarei et al., 2011	OCD HC	26/14 26/14	16.6 ± 1.5 16.5 ± 1.4	5.3 ± 3.4	11.2 ± 2.8	109.4 ± 12.5 110.8 ± 10.3 WASI	23/3/0 23/3/0	NA	9.8 ± 7.3 NA	19.5 ± 7.6 NA	CY-BOCS	SRRIs (*n* = 16)	sex, age, handedness, and IQ	NA	NA	NA
Gruner et al., 2012a, b	OCD HC	23/13 23/12	14.3 ± 2.1 14.2 ± 2.2	NA	NA	106.0 ± 15.1 106.8 ± 11.1	19/4/0 17/6/0	NA	NA	CY-BOCS obsessions: 13.09 ± 2.9 CY-BOCS compulsions: 13.78 ± 2.3 CY-BOCS total: 26.87 ± 4.5	K-SADS-PL	Psychotropic drug-na ¨ıve (*N* = 9) Past SSRI(*N* = 2) Current SSRI(*N* = 12)	sex, age, handedness, IQ	NA	CY-BOCS Multi- dimensional Anxiety Scale for Children Edinburgh Handedness Inventory Wechsler Abbreviated Scale of Intelligence executive functions: the Stroop Color Word Test, the Wisconsin Card Sorting Test (WCST-64), the Controlled Oral Word Association Test (COWAT), and the Trail Making Test	major depressive disorder (*N* = 4) anxiety disorders (*N* = 4) social anxiety disorder (*N* = 2) panic disorder (*N* = 2) attention deficit hyperactivity disorder (*N* = 5)
Jayarajan et al., 2012	OCD HC	15/8 15/8	14.13 ± 1.8 14.31 ± 2.1	1.40 ± 1.04	12.73 ± 1.87	NA NA	15/0/0 15/0/0	NA	NA	10.87 ± 3.76 10.60 ± 3.72	MINI-KID CY- BOCS	fluoxetine (*n* = 7) sertraline (*n* = 4) escitalopram (*n* = 2)	age, sex, handedness, and years of education	NA	NA	specific phobia (*N* = 2) major depression (*N* = 1) social phobia (*N* = 1) oppositional defiant disorder (*N* = 1) separation anxiety disorder (*N* = 1)
Silk et al., 2013	OCD HC	16/6 22/16	12.77 ± 2.8 11.24 ± 2.1	NA	NA	performance IQ = 100.3 ± 15.8 mean verbal IQ 95.6 ± 17.2 performance IQ = 104.7 ± 10.3 mean verbal IQ 102.7 ± 14.8	16/0/0 22/0/0	NA	NA	NA	CY-BOCS	Fluoxetine (*n* = 1) Sertraline & Ritalin (*n* = 1)	NA	NA	NA	GAD (*N* = 3)
Fitzgerald et al., 2014a, b	OCD HC	36/16 27/11	14.1 ± 2.9 14.7 ± 3.1	NA	7.30 ± 3.1	NA	NA	9.9 ± 6.6 2.6 ± 3.1	NA	Present: 16.7 ± 7.8 lifetime: 27.0 ± 7.0 HC: NA	SOCOBS CY-BOCS K-SADS PL MASC CDI	unmedicated(*n* = 18, 6 had previously taken a (SSRI) and 1 had taken a psychostimulant medicated (*n* = 18,fluoxetine (*n* = 10; augmented by aripiprazole in 1 patient and quetiapine in another); sertraline (*n* = 6); paroxetine (*n* = 1); and guanfacine (*n* = 1)	age, gender, handedness	MASC CDI	NA	OCD only (*N* = 9) 1 comorbidity (*N* = 15) 2 comorbidities (*N* = 12) (SAD, *N* = 3), social phobia (*N* = 2), (GAD, *N* = 7), specific phobia (*N* = 2), panic disorder (*N* = 2), major depression (*N* = 6), dysthymia (*N* = 1), tics (*N* = 11), impulse control disorder (*N* = 2), (ADHD, *N* = 2)
Rosso et al., 2014	OCD HC	17/11 19/13	14.06 ± 2.6 13.58 ± 2.1	5.24 ± 2.97	8.82 ± 3.36	NA	NA	8.94 ± 7.16 NA	NA	17.06 ± 8.17 NA	KSADS	unmedicated(*n* = 3) medicated(*n* = 14 – primary: antidepressants: selective serotonin reuptake inhibitors (*N* = 13) and tricyclic agents (*N* = 1) Secondary: stimulants (*N* = 4), mood stabilizers (*N* = 3), and benzodiazepines (*N* = 1))	age, gender, education	CDI	NA	OCD only (*n* = 9) GAD (*N* = 1), specific phobia (*N* = 2), agoraphobia (*N* = 1), major depressive disorder (*N* = 2), depression not otherwise specified (*N* = 2), oppositional defiant disorder (*N* = 1), ADHD (*N* = 2)
Lazaro et al, 2014	OCD HC	63/36 37/19	15.6 ± 2.1 15.8 ± 1.9	28.8 ± 24.1	13.1 ± 2.6	NA	NA	13.8 ± 9.6	NA	At time of MRI scan 17.8 ± 8.3 Maximum illness severity 27.1 ± 7.2	CY-BOCS OCI-CV CDI SCARED	SSRI(*n* = 53)	age, gender, estimated IQ	NA	NA	GAD (*N* = 13) (ADHD *N* = 6) (Anorexia nervosa *N* = 5) (Tic disorder *N* = 5) (Hypomania *N* = 2) (Major depressive disorder *N* = 2) (SAD *N* = 1) (Panic disorder *N* = 1) (Bulimia nervosa *N* = 1) (Oppositional defiant disorder *N* = 1) (Enuresis *N* = 1)
Gasso et al, 2015	OCD	54/30	15.7 ± 2.1	NA	13.3 ± 2.6	NA	NA	NA	NA	18.1 ± 8.7	CY-BOCS	48(SSRI)	–	–	NA	GAD (*N* = 13) ADHD(*N* = 6) Anorexia nervosa(*N* = 5) Tourette disorder(*N* = 4) Oppositional defiant disorder(*N* = 3) Hypomania(*N* = 2) Major depression disorder(*N* = 2) Panic disorder(*N* = 1) Bulimia nervosa(*N* = 1) Social phobia(*N* = 1)
White et al., 2015	EOS EOB OCD HC	43 (43)/6 13 (13)/13 17 (17)/6 29 (29)/13	17.0 ± 1.8 15.4 ± 2.1 16.2 ± 1.6 16.5 ± 2.0	NA NA NA NA	14.4 ± 1.6 14.1 ± 2.1 11.2 ± 3.3 NA	89.0 ± 16.1 94.3 ± 15.5 107.5 ± 13.0 106.9 ± 15.4 WASI	36/6/1 10/2/1 17/1/0 26/2/1	NA	NA	NA	DSM-IV K-SADS-PL	atypical (*n* = 35) / typical (*n* = 2) neuroleptics atypical neuroleptics (*n* = 10) SSRIs (*n* = 16) / Aripiprazole (*n* = 1)	Sex handedness	Age(patients with EOS being older than children with EOB (F1,53 = 7.7, P = 0.008)) IQ (with both patients with EOS (F1,68 = 21.7, P < 0.0001) and EOB (F1,39 = 5.6, P = 0.02) having lower IQ compared to the controls)	NA	NA
Ameis et al., 2016	ASD ADHD OCD HC	71/56 31/25 36/22 62/37	11.4 ± 3.4 10.3 ± 1.8 12.6 ± 2.6 10.8 ± 2.8	NA NA NA	NA NA NA	95 ± 19.7 103.4 ± 12.6 112.5 ± 17.1 112.5 ± 17.1	70/1/0 30/1/0 32/4/0 61/1/0	NA	NA	NA	CY-BOCS	29 13 13	Handedness	Age; sex; Child Behavior checklist (attention); Toronto Obsessive–compulsive Scale; social Communication Questionnaire; Adaptive Behavior Assessment System-II	Child Behavior checklist (attention); Toronto Obsessive–compulsive Scale; social Communication Questionnaire; Adaptive Behavior Assessment System- II	3 (2 ADHD) 23 (2 ASD, 1 OCD) 13 (6 ADHD)
Pagliaccio et al., 2020	OCD HC	28/14 27/14	12.14 ± 3.34 11.26 ± 3.23	NA	NA	106.96 ± 16.20 109.59 ± 12.14	NA	NA	NA	24.32 ± 5.14	CY-BOCS	NA	NA		NA	NA
Piras et al., 2021a, b, c	OCD HC	174/94 144/74	14.5 ± 2.3 14.3 ± 2.5	NA	13.1 ± 5.3	NA	NA	NA	NA	20.7 ± 7.8	DSM-IV MINI KSADS-PL YBOCS	Medicated (*N* = 112)	NA	NA	NA	Anxiety (*N* = 27) Major depression (*N* = 10)
Pagliaccio et al, 2021	OCD HC	1099/659 10584/5446	9.88 ± 0.6 9.91 ± 0.622	NA	NA	Total Cognition age-corrected T-scores: 100 ± 15	NA	NA	NA	CBCL OCS T-score: OCD 60.69 ± 9.22 HC 52.97 ± 5.2	K-SADS	859 unmedicated	Age Pubertal status	Sex parents marital status Parental education Parental income	NIH Toolbox OCD 97.3 ± 18.48 HC 100.7 ± 17.89	Any Depressive Disorder (*N* = 247) MDD (*N* = 137) Dysthymia (*N* = 6) Depression NOS (*N* = 117) Any Anxiety Disorder (*N* = 419) Separation Anxiety (*N* = 263) Social Anxiety (*N* = 477) GAD (*N* = 195) ADHD (*N* = 506) ODD/CD (*N* = 385) PTSD (*N* = 90)
Tikoo et al, 2021	TS TS + OCD OCD HC	16/15 14/10 11/7 12/3	9.7 ± 2.1 10.2 ± 2.1 10.7 ± 2.5 10 ± 1.2	NA	NA	All patients > 70	16/0/0 14/0/0 11/0/0 12/0/0	NA	NA	0.25 ± 0.7 16.4 ± 6.1 19.4 ± 7.5	CYBOCS	All patients were unmedicated	Age Sex	NA	WISC-III	Patients had no comorbidities
Grazioplene et al, 2022	HC	1208/568	14.19 ± 3.33	NA	NA	NA	NA	NA	NA	NA	GOASSESS for Obsessive–Compulsive Symptoms	NA	NA	NA	Penn Computerized Neurocognitive Battery	NA

Abbreviations: *HC* healthy controls; *OCD* obsessive–compulsive disorder; *NA* not assessed; *SOCOBS* Schedule for Obsessive–Compulsive and Other Behavioral Syndromes; *CY-BOCS* Children’s Yale–Brown Obsessive Compulsive Scale; *K-SADS PL* Kiddie-Schedule for Affective Disorders–Present and Lifetime Version; *CBCL* x Child Behaviour Checklist; *SAD* separation anxiety disorder; *GAD* generalized anxiety disorder; *ADHD* attention-deficit/ hyperactivity disorder; *SSRI* selective serotonin reuptake inhibitor; *CDI* Children’s Depression Inventory; *BDI* Beck Depression Inventory; *MASC* Multidimensional Anxiety Scale for Children; *KSADS* Kiddie Schedule for Affective Disorders and Schizophrenia; *EOS* Early-onset schizophrenia; *EOB* early-onset Bipolar affective disorder; *WASI* Wechsler Abbreviated Scale of Intelligence; *SSRIs* selective serotonin reuptake inhibitors; *EHI* Edinburgh Handedness Inventory; *SCARED* Screen for Childhood Anxiety Related Emotional Dis- orders; *CDI* Children’s Depression Inventory; *MINI-KID* Mini International Neuropsychiatric Interview-Kid; *K-SADS-PL* Schedule for Affective Disorders and Schizophrenia for School-Age-Children, Present and Lifetime Version; *MDD* major depressive disorder; *PTSD* post-traumatic stress disorder; *ODD* oppositional defiant disorder; *TS* Tourette syndrome; *WISC-III* the Wechsler intelligence scale for children III

**Table 2:** Overview of reviewed articles; DTI analysis and between-groups diffusion findings

Study	DTI analysis			Between-groups findings					
	Field Strength (T)	b value (s/mm2)	Method of analysis: Tracts/Regions studied	FA alterations	Higher MD (OCD > HC)	Higher AD (OCD > HC)	RD alterations	Other imaging findings	Clinical correlations
Zarei et al., 2011	1.5	1000	TBSS	OCD > HC: left inferior longitudinal fasciculus bilateral superior longitudinal fasciculus right inferior-fronto-occipital fasciculus bilateral cortico-spinal tract splenium of corpus callosum genu of corpus callosum bilateral forceps major bilateral forceps minor left cingulum right uncinate fasciculi	–	–	–	–	Symptom severity: FA: left uncinate fasciculi cortico-spinal tract superior longitudinal fasciculus forceps major forceps minor superior corona radiata genu of corpus callosum anterior thalamic radiation posterior limb of the internal capsule
Gruner et al., 2012	3	–	TBSS	OCD > HC: left dorsal cingulum bundle splenium of corpus callosum right cortico-spinal tract left inferior-fronto-occipital fasciculus	–	left dorsal cingulum bundle left inferior-fronto-occipital fasciculus	OCD < HC: left dorsal cingulum bundle splenium of corpus callosum right cortico-spinal tract left inferior-fronto-occipital fasciculus	–	Total obsessions on the CYBOCS in the subgroup of psychotropic drug-naive patients: FA: splenium of the corpus callosum Global executive functioning score and the response inhibition/cognitive control cluster score; response inhibition/ cognitive control domain score; better performance on the Stroop Color-Word Test and Trail Making; better response inhibition/cognitive control performance in the psychotropic drug-naive patients: FA: Left dorsal cingulum bundle
Jayarajan et al, 2012	3	800	TBSS	–	–	corpus callosum (splenium, genu and the body) right and left superior longitudinal fasciculus left inferior longitudinal fasciculus right and left cingulum bilateral anterior thalamic radiations bilateral anterior limb of the internal capsule left posterior limb of the internal capsule middle cerebellar peduncle	OCD > HC: genu of corpus callosum right and left superior longitudinal fasciculus right and left uncinate fasciculi bilateral anterior thalamic radiation bilateral inferior-fronto-occipital fasciculus left posterior limb of the internal capsule right superior cerebellar peduncle middle cerebellar peduncle right inferior cerebellar peduncle	–	–
Silk et al., 2013	3 T	1000	TBSS	–	–	Genu and splenium of corpus callosum	–	–	scores on the CBCL-OCS: negative correlation with AD: left cingulate superior longitudinal fasciculus bilateral posterior limbs of the internal capsule
Fitzgerald et al., 2014a, b	3 T	800	TBSS ROI: Anterior corpus callosum (genu, anterior body, and mid-body), anterior cingulum bundle, anterior limb of the internal capsule	OCD < HC: (effects of group): Genu↓ no main effects of age (group × age interaction effects): ↑ anterior corpus callosum anterior cingulum bundle anterior limb of the internal capsule	–	–	–	–	No effects of medication status or comorbidity were observed – gender: less FA in boys than in girls in genu of corpus callosum
Rosso et al., 2014	3 T	700	TBSS	OCD < HC: bilateral frontal and corpus callosum right cingulate and basal ganglia right posterior cerebral cortex right inferior frontal lobe(subcallosal cortex) right inferior frontal(orbitofrontal cortex) right thalamus right caudate, anterior internal capsule	–	–	OCD > HC: right frontal cortex and right body of corpus callosum	–	Lower age at onset was associated with decreased FA in right thalamus and with increased RD in the right body of corpus callosum
Lazaro et al, 2014	3	1000	VBA	OCD < HC: anterior region of the corpus callosum(genu of corpus callosum and the anterior portion of the body of corpus callosum)	anterior region of the corpus callosum anterior cingulate middle frontal gyri bilaterally right superior frontal gyrus anterior and posterior lobes and the pons left inferior frontal gyrus and left lentiform nucleus lingual gyrus of the occipital lobe	anterior region of the corpus callosum anterior cingulate middle frontal gyri bilaterally right superior frontal gyrus cerebellum (anterior and posterior lobes and the pons) left inferior frontal gyrus left lentiform nucleus lingual gyrus of the occipital lobe	OCD > HC: anterior region of the corpus callosum anterior cingulate middle frontal gyri bilaterally right superior frontal gyrus OCD > HC: Cerebellum (anterior and posterior lobes and the pons) left inferior frontal gyrus left lentiform nucleus lingual gyrus of the occipital lobe	–	presenting harm and checking symptoms: decreased FA: corpus callosum left anterior cingulate gyrus anterior region of the left caudate nucleus contamination and washing symptoms: decreased FA: left midbrain, lentiform nucleus insula thalamus increased MD, AD, and RD: anterior lobe of the cerebellum bilaterally pons
Gasso et al., 2015	3	1000	VBA	OCD < HC: corpus callosum	corpus callosum right and left anterior cingulate gyrus right and left medial frontal gyrus and superior frontal gyrus right cerebellum (anterior and posterior lobe) left cerebellum (culmen and lingual lobes) left inferior frontal gyrus and lentiform nucleus left lingual gyrus occipital lobe	–	–	–	MD values right anterior and posterior cerebellum: SLC1A1 rs3087879 (major allele homozygous) SLC6A3 rs4975646 (major and minor allele homozygous) NGFR rs2072446 NGFR rs734194 CDH9 rs6885387 left anterior and posterior cerebellum: DRD3 rs3773679(minor allele frequency) NGFR rs734194 (minor allele homozygous) lingual gyrus of the occipital lobe: CDH9 rs6885387 (heterozygous)
White et al., 2015	1.5 T	1000	TBSS	OCD < HC: Right Thalamic radiation	–	–	–	–	–
Ameis et al., 2016	3 T	1000	TBSS	OCD < HC: genu and splenium of corpus callosum cortico-spinal tract inferior longitudinal fasciulus inferior-fronto-occipital fasciculus arcuate fasciculus	–	–	–	–	adaptive functioning scores: FA: genu and splenium of corpus callosum cortico-spinal tract inferior longitudinal fasciulus inferior-fronto-occipital fasciculus arcuate fasciculus
Pagliaccio et al., 2020	3 T	1000	ROI NBS analysis	–	–	–	–	healthy > OCD differences in streamline count: left anterior cingulate cortex insular cortex thalamus putamen inferior, middle, and superior frontal sulci	–
Piras et al., 2021a, b, c	–	–	TBSS	In the pediatric cohort, patients showed no detectable FA abnormalities in any of the regions studied	–	–	–	Lower FA in adult OCD compared to HC in: genu and of corpus callosum posterior corona radiata posterior thalamic radiation sagittal stratum uncinate fasciculus	–
Pagliaccio et al, 2021	3	1000	TBSS	higher OCS scores related to lower FA in in the left superior cortico-striatal tract (particularly in the parietal portion of the left superior cortico-striatal tract)	–	–	–	Higher OCS related to altered functional connectivity, including weaker within dorsal attention network connectivity weaker dorsal attention-default mode anti-correlation Dorsal attention-default mode connectivity predicted OCS at 1-year	–
Tikoo et al, 2021	3	1000	TBSS	TS + OCD > HC: all three cerebellar peduncles OCD < HC: all three cerebellar peduncles	TS + OCD < HC: all three cerebellar peduncles OCD > HC: all three cerebellar peduncles	–	–	OCD patients, in comparison to HCs, exhibited decreased dentate nucleus functional connectivity with the right precentral gyrus, left postcentral gyrus, left inferior temporal gyrus, bilateral thalamus, and left crus II	In OCD patients, CYBOCS score positively correlated with dentate nucleus functional connectivity with the bilateral prefrontal cortex and left orbitofrontal cortex
Grazioplene et al, 2022	3	1000	ROI: right/left OFC pathway, right/left cingulum bundle, right/left uncinate fasciculus, right/left posterior thalamic radiation, right/left sagittal stratum, left superior corticostriatal tract, and genu, body, and splenium of the corpus callosum Whole-brain tractography	FA and general psychopathology in the right inferior longitudinal fasciculus (negative association) FA and general psychopathology × age effect in the right superior longitudinal fasciculus (positive association) FA and general psychopathology in the youngest quantile in distinct brain regions (positive association), and in the oldest (negative association) FA and Repetition/Checking in bilateral clusters of white matter in the centrum semiovale, including portions of the corpsus callosum and CST (negative association)	–	–	–	–	Fiber density and age in the younger age range (positive association) Fiber density and age in the highest age range (negative association) General psychopathology score and fiber density in a large region in the splenium of the corpus callosum (positive association) Bad Thoughts and fiber density in the dorsal splenium of the corpus callosum and in a portion of the left ascending corticospinal tract (positive association) Repetition/Checking and fiber cross-section in the splenium (positive association) Need for Symmetry and fiber density and cross-section in two regions of the corpus callosum (negative association) Age^2^ and Repetition/Checking and fiber cross-section and in the left external capsule in the youngest and oldest age groups (negative association) but no association in the middle age range

Abbreviation: *OCD* obsessive compulsive disorder; *HC* healthy control; *TBSS* tract-based spatial statistics; *ROI* Region-of-Interest; *CBCL-OCS* Child Behavior Checklist Obsessive–Compulsive Scale; *OCS* obsessive compulsive symptoms; *TS* Tourette syndrome; NBS: network-based statistics

Risk of bias assessment: We used the “Newcastle – Ottawa Quality Assessment Scale” (NOS) (Peterson et al., 2011) which is a widely used scale to assess the risk of bias in observational studies (or clinical trials) with ratings of biases arising from the selection, comparability, on a scale of 0–9 (Table 3).

**Table 3:**
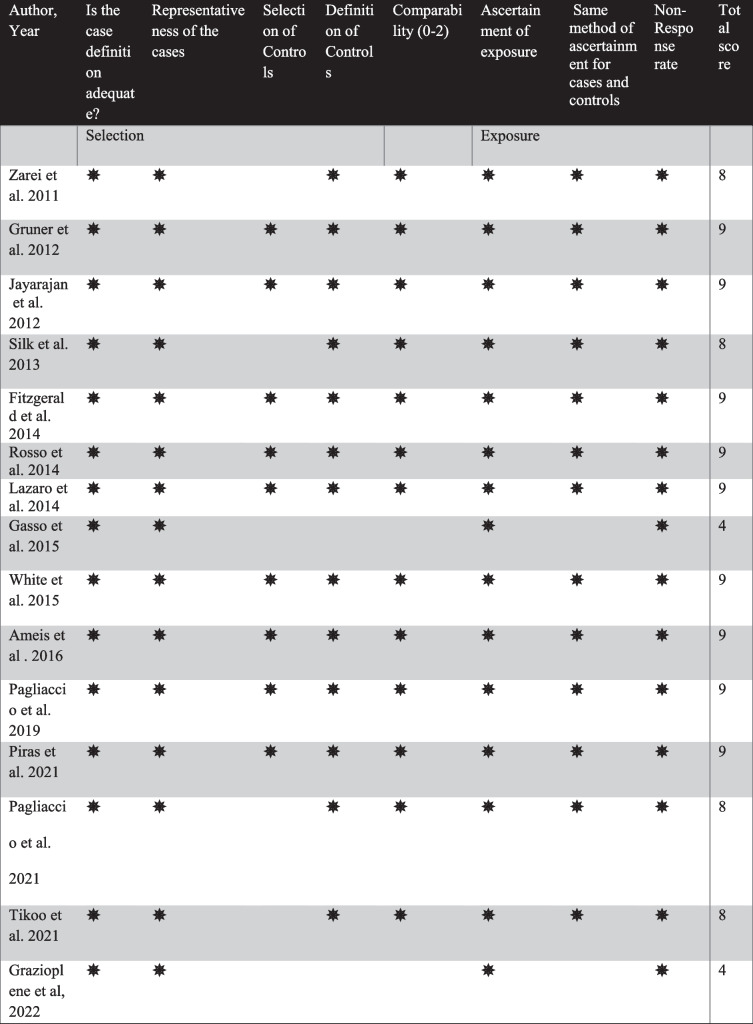
Newcastle–Ottawa Scale (NOS) risk of bias assessment of the included studies

## Results

We initially found 3634 articles, of which 626 were duplicates and therefore removed. We screened a total of 3008 articles by title and abstract to decide if these studies met any of the exclusion criteria. 105 studies were selected afterward, and full-text screening yielded 15 articles that were included in this review (Fig. 2).

All of the studies included in this review were assessed for selection, comparability, and exposure based on the Newcastle–Ottawa Scale (NOS) (Peterson et al., 2011). The median score for the included studies was 9 (4–9). Two studies focused on obsessive–compulsive symptoms and only included healthy participants with no control group thus their NOS score was low (Gasso et al., 2015; Grazioplene et al., 2022). Three studies lacked one score due to poor control selection (Pagliaccio et al., 2021; Tikoo et al., 2021; Zarei et al., 2011) and the other studies reached the complete NOS score.

## DTI findings in paediatric OCD patients

Studies revealed altered integrity of association fibers, including the cingulum, inferior longitudinal fasciculus, superior longitudinal fasciculus, inferior fronto-occipital fasciculus, uncinate fasciculus, and arcuate fasciculus; commissural fibers including corpus callosum, posterior limb of the internal capsule, thalamic radiation, corona radiata, and both forceps major and minor; and projection fibers including the corticospinal tract. Studies showed diverse changes in FA value in widespread regions of WM. Most studies demonstrated MD, AD, and RD values were increased in paediatric OCD patients compared to healthy controls. However, there was a report of lower RD in paediatric OCD patients compared to healthy controls in four WM areas: the left dorsal cingulum bundle, the splenium, the right corticospinal tract, and the left inferior fronto-occipital fasciculus (Gruner et al., 2012). There was a trend of FA reduction in paediatric OCD patients compared to healthy controls. However, two studies revealed higher FA in paediatric OCD patients than in HC in the left inferior longitudinal fasciculus, bilateral superior longitudinal fasciculus, right and left inferior fronto-occipital fasciculus, bilateral corticospinal tract, corpus callosum, splenium and genu, bilateral forceps major, bilateral forceps minor, left dorsal cingulum bundle and right uncinate fasciculus (Gruner et al., 2012; Zarei et al., 2011). Two studies did not show any WM tract alterations in paediatric OCD (Pagliaccio et al., 2020; Piras et al., 2021a, b, c).

The study of the ENIGMA group (Piras et al., 2021a, b, c) is the largest of its kind in terms of the number of cases included. Surprisingly, this study did not report any WM alterations in paediatric OCD. There are several methodological considerations that might have affected the result of this study. Firstly, meta-analysis although increases the power of the study by combining several smaller studies, only deals with the main effect and increases the homogeneity of the cohort, leading to the potential loss of a real effect which may be limited to a subpopulation of the cohort (Mavridis et al., 2018). For example, the rate of hoarding was almost twice as much in paediatric OCD (41%) in comparison to adult OCD (22%). Participants of smaller prospective studies are more precisely characterised, and the data is more accurately analysed. Secondly, the study used TBSS to obtain FA maps but did not perform voxel-wise comparison of FA skeletons. Group comparison was carried out using average FA value in 25 predefined tracts, extracted from an Atlas. This approached practically eliminate any chance of detecting regional changes within the tracts. Thirdly, AD, MD and RD were only assessed in the tracts that were showed to have significant group difference in FA. Fourthly, not all the studies included in the ENIGMA group study used the same method of analysis, some used TBSS and some used VBA. All these factors may potentially increase the homogeneity of the cohort leading to increased variability.

Two of the studies performed VBA analysis which could yield false positive results in the voxels close to the edge of WM (Gasso et al., 2015; Lazaro et al., 2014), and two other study used tractography technique to evaluate the structural connectivity (Grazioplene et al., 2022; Pagliaccio et al., 2020). The rest of the studies used the TBSS method. One study purely focused on cerebellar involvement (Tikoo et al., 2021).

Six studies showed that FA measures in the corpus callosum were significantly different in paediatric OCD compared to HCs. Two studies demonstrated that paediatric OCD patients had higher FA values in splenium and genu of the corpus callosum (Gruner et al., 2012; Zarei et al., 2011). FA tends to increase in the early puberty (Brouwer et al., 2012). Participants in one of our previous studies had a mean age of over sixteen years (Zarei et al., 2011). The other four studies showed that FA was significantly lower in paediatric OCD in anterior corpus callosum regions and also in splenium and genu of the corpus callosum (Ameis et al., 2016; Fitzgerald et al., 2014a, b; Lazaro et al., 2014; Rosso et al., 2014).

MD changes in the corpus callosum were prominent in merely one study; Lazaro and colleagues found increased MD in the anterior region of the corpus callosum (Lazaro et al., 2014). AD values were altered in the corpus callosum tract in three studies. These alterations were in the genu, body, splenium, and anterior parts of the corpus callosum (Jayarajan et al., 2012; Lazaro et al., 2014; Rosso et al., 2014). RD changes were manifested in the corpus callosum in four studies. These changes appeared across the corpus callosum in splenium, genu, body, or the anterior part of the corpus callosum. These parts showed higher RD in paediatric OCD in comparison with healthy controls (Gruner et al., 2012; Jayarajan et al., 2012; Lazaro et al., 2014; Rosso et al., 2014). Lazaro et al. study had the largest sample size and found changes in all diffusivity metrics in the corpus callosum (Lazaro et al., 2014). They highlighted the role of the corpus callosum in OCD neurobiology. Whole brain studies which used TBSS analysis in OCD patients found altered corpus callosum WM integrity with two exceptions (Piras, et al., 2021a, b, c; White et al., 2015). White et al. used a 1.5 T scanner; a weaker magnet that would give less detailed images. Interestingly, they were the only study to find FA reduction in the right thalamic radiation in OCD patients compared to controls and this was their only significant finding.

Based on the included studies, forceps minor and forceps major are the other commissural fibers involved in paediatric OCD. Forceps minor connects the bilateral frontal lobes which carries orbitofronto-striatal fibers, and forceps major connects the bilateral occipital lobes and is not relevant to affective or executive circuits. We previously found that both of these tracts have higher FA in paediatric OCD compared to the healthy controls (Zarei et al., 2011). This study is also unique as it found a relationship between white and grey matter changes in OCD.

Six studies showed cingulum involvement in paediatric OCD. The left dorsal cingulum bundle showed higher FA in paediatric OCD. There was also a decreased FA in the anterior cingulum bundle of paediatric OCD patients compared to healthy controls (Fitzgerald et al., 2014a, b; Gruner et al., 2012; Zarei et al., 2011). Significantly higher MD and AD and lower RD were observed in the left dorsal cingulum bundle of paediatric OCD patients compared to healthy controls (Gruner et al., 2012; Jayarajan et al., 2012; Lazaro et al., 2014). Pagliaccio et al. showed decreased streamline count in the left anterior cingulate cortex of OCD patients (Pagliaccio et al., 2020). Two studies found uncinate fasciculus DTI changes in paediatric OCD. We previously found increased FA while Jayarajan and colleagues. Reported increased RD (Jayarajan et al., 2012; Zarei et al., 2011). One study showed lower FA in the arcuate fasciculus in paediatric OCD compared to healthy controls (Ameis et al., 2016). One study showed FA increase (Zarei et al., 2011) and another one showed AD increase in paediatric OCD in the superior longitudinal fasciculus, and inferior longitudinal fasciculus (Jayarajan et al., 2012). They also found bilateral RD increases in the superior longitudinal fasciculus in paediatric OCD. Four studies found alterations in inferior-fronto-occipital fasciculus integrity. These changes included increased FA (Gruner et al., 2012; Zarei et al., 2011) and decreased FA (Ameis et al., 2016), increased (Jayarajan et al., 2012), and decreased RD (Gruner et al., 2012), and increased AD values (Gruner et al., 2012) in paediatric OCD. Among projection fibers, cerebellar peduncles and corticospinal tract were found to be involved in paediatric OCD. While corticospinal tract is a part of “Executive” dorsolateral prefronto-striatal circuit, cerebellar peduncles were not previously attributed to OCD pathophysiology (Menzies et al., 2008a, b).

Four of the studies included in this review showed corticospinal tract alterations in paediatric OCD patients. Studies by Gruner and colleagues as well as our own showed that patients with paediatric OCD displayed higher FA values compared to healthy controls in the corticospinal tract (Gruner et al., 2012; Zarei et al., 2011). However, Ameis and colleagues showed lower FA in paediatric OCD patients versus healthy controls in the corticospinal tract (Ameis et al., 2016). Pagliaccio and colleagues in a large sample study found that paediatric OCD patients had lower FA in the lower parietal superior part of the corticospinal tract compared to HC (Pagliaccio et al., 2021). There were no reports of MD or AD changes in the corticospinal tract in paediatric OCD.

Three studies showed cerebellum WM or its peduncles are involved in paediatric OCD. Lazaro and colleagues showed higher MD, AD, and RD values in the anterior and posterior lobes of the cerebellum and pons (Lazaro et al., 2014). Gasso and colleagues found higher MD in the anterior and posterior lobe of the right cerebellum and culmen and lingual lobes of the left cerebellum (Gasso et al., 2015). Tikoo and colleagues, (Tikoo et al., 2021) showed that paediatric patients with Tourette’s syndrome and OCD had higher FA in all three cerebellar peduncles but patients with mere OCD diagnosis, had lower FA in all three cerebellar peduncles compared to healthy controls. The importance of cerebellum in OCD has also been highlighted by functional MRI study that showed decreased functional connectivity of dentate nucleus with the left crus II of the cerebellum. Among all the above studies the most consistent findings were changes in the cingulum tract, inferior-fronto-occipital tract, and corticospinal tract. Most studies used TBSS analysis, except one which reported higher MD in the cingulum tract (Lazaro et al., 2014) and another which reported a lower streamline count in the left anterior cingulate cortex of OCD patients (Pagliaccio et al., 2020).

## Sex-specific pattern and DTI parameters

The study by Fitzgerald et al. investigated the effects of age and sex on FA in each group of paediatric OCD patients and healthy controls in the corpus callosum, cingulum bundle, and anterior limb of the internal capsule. Their data showed that the increase of FA in patients compared with healthy controls is related to age. This relationship was much more prominent for girls in the anterior cingulum bundle. Moreover, they found lower FA in the genu of the corpus callosum in boys compared to girls (Fitzgerald et al., 2014a, b). Gasso et al. found that gender was linked with MD values in a cluster involving the inferior frontal gyrus and lentiform nucleus in OCD patients (Gasso et al., 2015).

## Clinical correlations and DTI parameters

WM FA of many regions revealed a significant positive correlation with the symptoms’ severity. These regions included the left uncinate fasciculus, corticospinal tract, superior longitudinal fasciculus, forceps major, forceps minor, superior corona radiata, splenium, anterior thalamic radiation, and posterior limb of the internal capsule. We previously showed a significant negative correlation between the left hippocampal cingulum bundle adjacent to the entorhinal cortex bilaterally and the symptom severity (Zarei et al., 2011). However, Gruner et al. study did not find any significant correlations between symptom severity and FA values of the left dorsal cingulum bundle, splenium of corpus callosum, right corticospinal tract, and the left inferior fronto-occipital fasciculus. These fibers showed higher FA in patients compared to healthy controls (Gruner et al., 2012).

One study showed that total obsession score was significantly associated with higher FA in the splenium of corpus callosum. (Gruner et al., 2012) In addition they found that executive functions had a significant direct correlation with the left dorsal cingulum bundle FA. Presenting harm and checking symptoms were accompanied by decreased FA in corpus callosum, left anterior cingulate gyrus, and anterior region of the left caudate nucleus, while contamination and washing symptoms correlated with decreased FA in the left midbrain, lentiform nucleus, insula, and thalamus. Adaptive functioning scores were positively correlated with FA in the genu and splenium of the corpus callosum, corticospinal tract, inferior longitudinal fasciculus, arcuate fasciculus, and inferior fronto-occipital fasciculus (Ameis et al., 2016). Adaptive functioning scores positively correlated with FA among patients, particularly in genu and splenium of corpus callosum, as well as in the corticospinal tract, inferior longitudinal fasciculus, arcuate fasciculus, inferior fronto-occipital fasciculus (Ameis et al., 2016).

Gruner et al. observed FA alterations in the left cingulum bundle associated with total obsessions scores and executive functioning scores (Gruner et al., 2012). Larazo and colleagues’ findings were in line with the Gruner study (Lazaro et al., 2014). They found that the left anterior cingulate gyrus changes correlated with presenting harm and checking symptoms.

As measured in the Child Behaviour Checklist-Obsessive Compulsive Scale (CBCL-OCS), the severity of symptoms in patients had a significant negative correlation with AD in the left cingulum bundle, superior longitudinal fasciculus, and bilateral posterior limb of internal capsule (Silk et al., 2013). A study showed that MD values in the right and left, anterior and posterior cerebellum were significantly correlated with specific alleles and single-nucleotide polymorphisms in paediatric OCD, but the anterior lobe of the cerebellum and pons were correlated with contamination and washing symptoms (Lazaro et al., 2014). Grazioplene et al. found that FA in the inferior longitudinal fasciculus was negatively associated with general obsessive–compulsive symptoms psychopathology scores, but this association was positive for the superior longitudinal fasciculus (Grazioplene et al., 2022). Interestingly these FA changes had a trend of a positive association in the youngest quantile and a negative association in the oldest. a They also found a negative association between FA and Repetition/Checking symptoms in the corpus callosum and cortico-spinal tract.

## Medication and DTI parameters

In Zarei et al. and Gruner et al. study, ROI analysis of specific regions showed no difference between medicated and unmedicated patients (Gruner et al., 2012; Zarei et al., 2011). Moreover, no significant effects of medication on FA value were found in Fitzgerald et al. study (Fitzgerald et al., 2014a, b). While Piras et al. did not find any WM changes in paediatric OCD, they reported that lower FA in the sagittal stratum of adult OCD patients was associated with a higher percentage of medicated patients which is an important confounder in the study (Piras et al., 2021a, b, c).

## Genetic and DTI parameters

Gasso and colleagues investigated the association between the MD value of WM structure and specific genetic polymorphisms (Gasso et al., 2015). Results showed the existence of an association between specific polymorphisms in genes of glutamatergic, dopaminergic, and neurodevelopmental pathways with MD value especially discovered in the region of right and left anterior and posterior cerebellar lobes and in the lingual gyrus of the occipital lobe. As mentioned, anterior and posterior lobes of the cerebellum, pons, and lingual gyrus had significantly higher MD, AD, and RD values compared with HCs, suggesting an involvement of these regions in the pathophysiology of the paediatric OCD (Lazaro et al., 2014).

Various polymorphisms in OCD were reported in glutamate transporter gene (rs3087879 (SLC1A1)), dopamine transporter gene (rs4975646, SLC6A3), dopaminergic receptor D3 (rs3777679, DRD3), nerve growth factor receptor gene (rs734194, rs2072446, NGFR) and the cadherin 9 gene (rs6885387, CDH9). Rs3087879 polymorphism of SLC1A1 gene had a significant correlation with higher MD value in paediatric OCD patients, especially in those with major allele homozygous (GG) for SLC1A1, rs3087879. The association of two polymorphisms of SLC6A3 rs4975646 and DRD3 rs3773679 with the MD value of WM also supported the involvement of the dopaminergic system. They also showed polymorphisms of NGFR, rs734194 and rs2072446, and CDH9 rs6885387 in association with WM microstructure. However, no association between these polymorphisms was found with FA.

These results show that there are several dopamine-related polymorphisms and glutamate-related polymorphisms linked to OCD and imply a polygenic model of OCD in which several genes contribute subtly and gradually to the likelihood of developing the condition. Interestingly, these polymorphisms are related to WM alterations. These findings highlight the crucial roles of dopamine and glutamate and WM integrity in the pathophysiology of OCD and support the participation of cortico-striatal-thalamic-cortical bundles in this process. They also support the participation of tracts outside the orbitofronto-striatal circuit including the cerebellum and occipital lobe.

## Models of OCD and DTI parameters

Studies included in this review support the classic model of orbitofronto-striatal circuit alterations in OCD, however, it is insufficient in explaining all OCD WM changes. These fifteen studies reported widespread alterations in WM tracts that are beyond orbitofronto-striatal circuit. They also reported the involvement of tracts in the temporal, parietal, and occipital lobes. Association fibers that anatomically connect areas of the brain classically known to be involved in OCD include the cingulum, uncinate fasciculus, and arcuate fasciculus. However, the superior longitudinal fasciculus, inferior longitudinal fasciculus, and inferior-fronto-occipital fasciculus also showed altered integrity in paediatric OCD (Ameis et al., 2016; Gruner et al., 2012; Jayarajan et al., 2012; Zarei et al., 2011). Overall, paediatric OCD patients had alterations in brain WM structure in three distinct networks: the first, involving the orbitofrontal circuits, the anterior cingulate bundle, and temporal poles; the second, including the postcentral and lingual gyri WM connections, and the third comprising a circuit made by connections between the thalamus and occipital regions.

Several structural alterations in the WM connecting the orbitofrontal cortex, anterior cingulate cortex, thalamus, and caudate nucleus in the included studies supported the classic orbitofronto-subcortical circuits model (Ameis et al., 2016; Fitzgerald et al., 2014a, b; Gasso et al., 2015; Lazaro et al., 2014; Piras et al., 2021a, b, c; Silk et al., 2013). OCD phenomenology can be explained by functional and anatomical activity in the orbitofrontal circuits. The caudate serves as a gate for the limbic and frontal cortices, the anterior cingulate cortex as an activity monitor and regulator, the orbitofrontal cortex as a monitor of proper conduct in social life, and the thalamus as an information filter (Nakao et al., 2014).

Structural changes outside the orbitofrontal circuits comprised areas on the dorsolateral frontal and parietal lobes that may be thought to reflect the dorsolateral prefronto-striatal circuit that involves cognitive networks like spatial or attentional cognition. Posterior areas, including the WM tracts in the parietal and occipital lobes and cerebellum, also displayed altered integrity (Gruner et al., 2012; Jayarajan et al., 2012; Pagliaccio et al., 2020, 2021; Tikoo et al., 2021). These regions are involved in cognitive tasks (Nakao et al., 2014). OCD was likely to have a complex pathophysiological condition if both the orbitofronto-striatum and prefronto-limbic-posterior circuits were involved.

Interestingly, studies observed that different paediatric OCD dimensions were linked to quite unique components inside and outside the frontostriatothalamic circuit. For instance, considering contamination/washing-related stimuli, patients showed substantially lower FA than controls in the midbrain, lentiform nucleus, insula, thalamus, and higher MD, AD, and RD in the cerebellum and pons, whereas when facing presenting harm and checking symptoms, patients showed significantly lower FA in the corpus callosum, cingulate gyrus, and caudate nucleus (Gruner et al., 2012; Lazaro et al., 2014).

The relationship between clinical symptoms, cognitive functions, and the brain may be shown by combining neuropsychological and neuroimaging approaches.

## Discussion

In this work, we conducted a systematic review of DTI investigations to study WM structure patterns in paediatric OCD patients. We found that several subnetworks of the WM in OCD patients are significantly disrupted, especially the networks that connect the medial orbitofrontal regions, the thalamus, the temporal poles, and the occipital regions.

Fifteen studies were included in this review. The majority of the studies were single-center case–control studies and included OCD groups and healthy controls. Two studies recruited healthy individuals with and without OCS (Grazioplene et al., 2022; Pagliaccio et al., 2021). These two studies were adequately powered and looked at the neural changes in the early stages of the disease. Three studies included an additional group of patients besides OCD and healthy controls. These three studies suggested that DTI is useful for the evaluation and comparison of white matter pathology (Ameis et al., 2016; Tikoo et al., 2021; White et al., 2015). Studying WM microstructural changes to characterize functional connectivity changes has a potential impact on clinical decision-making. Three studies investigated structural connectivity along with other imaging techniques (Pagliaccio et al., 2021; Tikoo et al., 2021; Zarei et al., 2011).

Many studies in the paediatric OCD population found diffuse WM alterations predominantly in the corpus callosum, cingulum, arcuate fasciculus, uncinate fasciculus, inferior longitudinal fasciculus, superior longitudinal fasciculus, inferior fronto-occipital fasciculus, corticospinal tract, forceps minor and major and the cerebellum. WM tracts connecting the prefrontal cortex, striatum, globus pallidus, and thalamus are primarily disrupted in paediatric OCD, however, there are WM changes beyond these tracts. Neural tracts connecting fronto-occipital lobes, occipito-temporal lobes, and cerebellum to the brain stem as well as corpus callosum are also involved in paediatric OCD. These inconsistent findings in these studies might reflect differences in methodological approaches particularly DTI analysis or be part of the pathophysiology of the disease spectrum.

Corpus callosum is heavily involved in the lateralization of sensorimotor and cognitive brain processes (Hoptman & Davidson, 1994; van der Knaap & van der Ham, 2011; Walterfang & Velakoulis, 2014). Studies using DTI to investigate how FA and MD values change during corpus callosum development showed that increased FA and decreased MD correlated with age (Barnea-Goraly et al., 2005; Bashat et al., 2005; Lebel et al., 2010; Schmithorst et al., 2008). Longitudinal DTI studies may be the best way to assess changes in the corpus callosum where neurodevelopmental changes are of particular interest.

The cingulum bundle connects frontal, parietal, and medial temporal lobes and basal ganglia with the cingulate gyrus (Bubb et al., 2018) and is thought to play an important role in the emotional and social cognition adjustment (Fitzsimmons et al., 2020), two types of behaviour that are often affected in OCD. Changes in WM integrity of the frontal and parietal lobes are also of relevance to OCD behaviour. These lobes are directly connected and play an important role in sensorimotor integration, which feeds directly into judgment and decision making. Involvement of other major pathways such as superior longitudinal fasciculus, inferior longitudinal fasciculus, superior fronto-occipital fasciculus, inferior fronto-occipital fasciculus, and the uncinate fasciculus is also reported. These pathways are primate-specific WM tracts connecting almost the entire cerebral cortex (Hua et al., 2008).

The corticospinal tract mainly contains pyramidal tracts that control voluntary muscle movements (Lemon & Griffiths, 2005). Corticospinal tract alterations in paediatric OCD patients were observed in some studies (Ameis et al., 2016; Gruner et al., 2012; Zarei et al., 2011). Involvement of this tract might be relevant to soft motor signs that have been frequently observed in OCD and related disorders (Bolton et al., 1998; Dhuri & Parkar, 2016; Ekinci & Erkan Ekinci, 2020; Malhotra et al., 2017). Cerebellar involvement in OCD was reported in three studies (Gasso et al., 2015; Lazaro et al., 2014; Tikoo et al., 2021). The cerebellum's role in the pathogenesis of a number of neuropsychiatric illnesses has attracted growing interest in recent years (Haghshomar et al., 2022). Previous studies have shown that the cerebellum may be crucial in the pathogenesis of OCD, as evidenced by OCD patients' aberrant spontaneous cerebellar activity and impaired functional connectivity between the cerebellum and the cortico-striato-thalamo-cortical circuit (Zhang et al., 2019).

OCD in children and adolescents is likely to have a neurodevelopmental basis. This is supported by DTI studies in paediatric OCD which showed various changes in WM integrity (Fitzgerald et al., 2014a, b; Gruner et al., 2012; Zarei et al., 2011). In addition, paediatric OCD was associated with increased thalamic and striatal volume (Van den Heuvel et al., 2022). It appears that in the process of maturation and growth the risk of psychiatric disorders increases (Paus et al., 2008). In several white-matter areas, DTI investigations show an age-related decline in the directionality and an increase in the magnitude of water diffusion. Such alterations in DTI-derived metrics may signify that axons and/or their myelin sheaths are still maturing and changes in the myelination, are prominent in this period (Schmithorst et al., 2002). The most widely accepted explanation for the anatomical findings in the adolescent brain includes alterations in synapse pruning and myelination (Snook et al., 2005). Changing levels of hormones occur throughout development, and steroid hormones have an impact on neuronal activity and morphology (Sisk & Foster, 2004). Notably, Grazioplene showed WM fiber density and age have a positive association in the younger age range and a negative association in the higher age range. They also found that FA changes in distinct areas of the brain have positive associations in the younger age quantile and negative associations in the older age quantile. This highlights the role of maturation in WM alterations and can explain contradictory results of FA alterations in paediatric OCD patients. Besides the maturation process, paediatric OCD patients experience a longer disease duration due to an early onset. Piras and colleagues found that FA reduction in the sagittal striatum correlated with OCD disease duration (Piras et al., 2021a, b, c). The latter is contrary to other studies including those in this review. The reason for this inconsistency might be methodological in origin. Studies included in this review were mostly based on TBSS analysis. This method directly maps diffusion values from each subject onto a reference skeleton for group comparison, building a white-matter skeleton, which is restricted to the center of WM pathways, in order to minimize the possible misalignment that may occur in voxel-based whole-brain analysis (Smith et al., 2006). The analysis of multi-subject diffusion imaging investigations is enhanced by TBSS in terms of sensitivity, objectivity, and interpretability. Besides different imaging protocols, clinical characterization of the cohorts and comorbidities may also be important confounding variables (Table 1) contributing to inconsistent findings.

Sex differences is also an important factor: there are differences in FA map of female vs male brain (Schmithorst et al., 2008). The interaction between sex and OCD subtypes is also a factor (den Braber et al., 2013), particularly that OCD is more common in males than females in childhood, but this propensity reverses in adulthood.

Another explanation for inconsistent results of DTI studies on paediatric OCD is the heterogeneity of OCD symptoms. OCD is a highly heterogeneous mental illness and individuals with the same diagnosis of OCD might come with completely different, non-overlapping obsessions or/and compulsions characteristics. There is a great variability in symptoms and severity of OCD, measured by the Yale-Brown Obsessive–Compulsive Scale (Y-BOCS) score. Mild symptoms are between 0–13, moderate 14–25, moderate-severe 26–34, and severe symptoms by 35–40 (Storch et al., 2015). Y-BOCS values in the DTI studies were between 10–28. Most patients had various and multiple obsessive and/or compulsive symptoms (OCS) and only a minority were monosymptomatic. A previous meta-analysis identified four OCS dimensions: contamination and washing; symmetry and arranging; banned ideas, and checking; and hoarding (Bloch et al., 2008).

We found that each OCS dimension might be connected to a particular neurobiological substrate. Based on the studies in this review contamination and washing-related symptoms were related to WM alterations within and also outside the frontostriatothalamic circuits while checking symptoms were associated with the main altered tracts in OCD including the corpus callosum and cingulate gyrus. Recent fMRI studies utilizing neuropsychological tasks during fMRI have shown a connection between cognitive impairment and clinical symptoms in OCD (Nakao et al., 2014).

A functional MRI study showed different patterns of cortical activation in relation to different categories of OCD symptoms; Mataix-Cols found that OCD patients showed significantly greater activation than controls in the thalamus, putamen/globus pallidus, and dorsal cortical regions when exposed to contamination and washing-related stimuli. In contrast, patients showed significantly greater activation than controls in the right caudate nucleus and bilateral ventromedial prefrontal regions when exposed to banned ideas and checking-related stimuli, (Mataix-Cols et al., 2008). Van den Heuvel et al. sought to examine variations in the volumes of different brain regions' white and grey matter and came to the conclusion that people with symmetry dimension symptoms had a smaller right motor cortex volume (Van Den Heuvel et al., 2009). Similar to this, Alvarenga et al. found that those with higher aggressiveness ratings had larger lateral parietal cortex sizes in both hemispheres, whereas people with higher sexual/religious dimension volumes had larger insula volumes in both hemispheres (Alvarenga et al., 2012).

This dimensional model of OCD has its limitations. Phenotypic data is used to build this model. Even if the stated symptomatology is of utmost significance in psychiatry, it might be deceptive to categorize people just by their symptoms. Different processes underlie the symptoms of OCS. Some large neuroimaging studies using mega-analysis approaches have failed to identify different neuroanatomical correlations for each OCS dimension (Boedhoe et al., 2017, 2018). This issue needs to be addressed in future studies.

Another factor is the effect of long-term pharmacotherapy, which may affect DTI measures (Insel et al., 2008; Wang et al., 2013). The majority of the patients in this review were on SSRI treatment. SSRIs were shown to affect diffusion measures (Seiger et al., 2021). SSRIs may also affect brain development by increasing Brain-Derived Neurotrophic Factor (Hunsberger et al., 2009), through its effect on oligodendrocytes (Xiao et al., 2010), and astrocytic glycogenolysis (Sijens et al., 2008). These changes may potentially alter diffusivity coefficients. Some paediatric patients with severe OCD were on antipsychotics. These drugs may also affect DTI measures, for example by reducing the number of glial cells and myelination process (Alexander et al., 2011; Konopaske et al., 2008). Clearly, the effect of medication could be a potential source of variability in DTI studies in paediatric OCD. What makes the effect of drugs on DTI measures even more complex is that this effect is unpredictable and variable between diseases and individuals (Sagarwala & Nasrallah, 2020). Taken together the exact effects of medication on WM changes are not fully understood and future studies should take this effect into account.

Finally, the result of this systematic review is limited by the low number of studies included, as well as differences in study design, imaging protocol, symptomatology, small sample sizes, and image analysis approach. A large prospective longitudinal study using multimodal structural and functional imaging methods from early childhood well into adulthood in a well-powered and clinically characterized cohort is required to understand the neural substrates of OCD and its relationship with growth and development. As studies show FA changes vastly during maturation, a recommendation for a future study is to investigate WM changes in distinct age groups of paediatric OCD patients.

## Conclusion

DTI is a useful tool for a deeper understanding of microstructural changes in the brain particularly in the WM. DTI studies of children with OCD demonstrated altered integrity in various anatomical connections. This technique together with other neuroimaging methods may play a vital role in our understanding of OCD. However, various factors affect the result of DTI analysis and therefore its interpretation should be considered with caution and with a full understanding of methodological issues and in the context of clinical information.

## Supplementary Information

Below is the link to the electronic supplementary material.

Supplementary file1 (DOCX 25 KB)

## Data Availability

Not applicable.
